# Genome-wide analysis of long non-coding RNAs unveils the regulatory roles in the heat tolerance of Chinese cabbage (*Brassica rapa* ssp.chinensis)

**DOI:** 10.1038/s41598-019-41428-2

**Published:** 2019-03-21

**Authors:** Aihua Wang, Jihong Hu, Changbin Gao, Guanglong Chen, Bingcai Wang, Chufa Lin, Liping Song, Yi Ding, Guolin Zhou

**Affiliations:** 1 Wuhan vegetable research institute, Wuhan Academy of Agricultural Science & technology, Wuhan, 430345 China; 2 Oil Crops Research Institute of the Chinese Academy of Agricultural Sciences, Key Laboratory of Biology and Genetic Improvement of Oil Crops, Ministry of Agriculture, Wuhan, 430062 China; 3 State Key Laboratory of Hybrid Rice, College of Life Sciences, Wuhan University, 430072 Wuhan, China

## Abstract

Long non-coding RNAs (lncRNAs) mediate important epigenetic regulation in various biological processes related to the stress response in plants. However, the systematic analysis of the lncRNAs expressed in *Brassica rapa* under heat stress has been elusive. In this study, we performed a genome-wide analysis of the lncRNA expression profiles in non-heading Chinese cabbage leaves using strand-specific RNA-sequencing. A total of 4594 putative lncRNAs were identified with a comprehensive landscape of dynamic lncRNA expression networks under heat stress. Co-expression networks of the interactions among the differentially expressed lncRNAs, mRNAs and microRNAs revealed that several phytohormones were associated with heat tolerance, including salicylic acid (SA) and brassinosteroid (BR) pathways. Of particular importance is the discovery of 25 lncRNAs that were highly co-expressed with 10 heat responsive genes. Thirty-nine lncRNAs were predicted as endogenous target mimics (eTMs) for 35 miRNAs, and five of them were validated to be involved in the heat tolerance of Chinese cabbage. Heat responsive lncRNA (TCONS_00048391) is an eTM for bra-miR164a, that could be a sponge for miRNA binding and may be a competing endogenous RNA (ceRNA) for the target gene NAC1 (Bra030820), affecting the expression of bra-miR164a in Chinese cabbage. Thus, these findings provide new insights into the functions of lncRNAs in heat tolerance and highlight a set of candidate lncRNAs for further studies in non-heading Chinese cabbage.

## Introduction

Heat stress seriously affects plant growth and development, and is a major limiter of crop productivity worldwide^1^. Metabolism, physiology and development were changed in response to heat stress. At the molecular level, the dramatic reprogramming of gene expression was modified upon heat stress^1,2^. After exposure to high temperatures, the tolerant plants can survive and induce genes involved in direct protection from stress, including the synthesis of detoxifying enzymes, transporters, and genes that encode regulatory proteins such as transcription factors and protein kinases^3^. Plant tolerance to heat stress involves various aspects to activate stress protection systems and maintain cell growth, photosynthesis as well as cell membrane stability^4^.

Plants use a variety of strategies to cope with environmentally induced stresses, in which phytohormones play important roles^5^. The induction of stress-responsive metabolites and heat shock proteins (HSPs) as well as hormone homeostasis has been reported to be correlated with tolerance to heat stress in plants^6^. Several plant hormones including abscisic acid (ABA), salicylic acid (SA) and ethylene increase their levels under heat stress, while other hormones decrease, such as auxin, cytokinin and gibberellic acids (GAs)^7^. ABA involvement in biochemical pathways is essential for survival under heat-induced desiccation stress^4^. In addition, brassinosteroids (BRs) have recently been reported to confer thermotolerance in tomato and oilseed rape (*Brassica napus*)^8^.

Recently, non-coding RNAs are emerging as important regulators of gene expression in plant development and in response to environmental stimuli. Among them, long non-coding RNAs (lncRNAs) are diverse classes of transcripts longer than 200 nt without or with little protein-coding potential^9^. In plants, the majority of lncRNAs are transcribed by RNA polymerase II, while, some lncRNAs are produced by RNA polymerase III or RNA polymerase IV/V^10,11^. LncRNAs have been documented to serve as precursors of miRNAs and other small RNAs, or as miRNA target mimics^12^. Accumulating evidence has shown that miRNAs involved in heat stress^9^. In *Arabidopsis*, miR164 was up-regulated and its target NAC1 was suppressed under high temperature^13^. Overexpression of miR159 led to the down-regulation of its target *GAMYB* and to decrease plant heat tolerance in wheat^14^. Recently, accumulating evidence has suggested that lncRNAs play important roles in plant stress responses^15,16^. Two lncRNAs (TalnRNA27 and TalnRNA5) which are miRNA precursors in wheat, have been reported to be up-regulated under heat stress^17^. In *P*. *simonii*, the expression level of PsiLncRNA00268512 was dynamic in response to heat stress^18^. Using the sensitive rice genotype, 2580 lncRNAs were identified and the ABA signaling pathway was enriched in response to Cd stress^19^. Using strand-specific RNA-seq, 16 of the 682 lncRNAs were identified to act as miRNA mimics, including cold-repressive lincRNA159 which is the target mimics of miR164 in cassava^16^. In *B*. *rapa*, systematic identification of lncRNAs during pollen development and fertilization revealed that 47 *cis*-acting lncRNAs and 451 *trans*-acting lncRNAs highly co-expressed with their target protein-coding genes^20^.

As one of the subspecies of *Brassica rapa* L., non-heading Chinese cabbage (*B*. *rapa* ssp. chinensis) is an economically and agriculturally significant leafy vegetable which is cultivated extensively in the world^21^. The adaptable growth temperature for this Chinese cabbage ranges from 18 °C to 22 °C. However, the production of non-heading Chinese cabbage is usually impaired by heat stress in many regions of China^22,23^. Studies on gene expression and the regulation of the responses to heat stress in Chinese cabbage are essential for breeding heat-resistant cultivars. In recent years, dozens of studies have been reported to elucidate of the mechanisms of heat-tolerance in Chinese cabbage^21–24^. Using heat-sensitive and heat-tolerant varieties of non-heading Chinese cabbage, the dynamic changes in genes were revealed in response to high temperature^23^. In Chinese cabbage, heat-responsive miRNA nat-siRNAs and chloroplast small RNAs (csRNAs), were identified to be highly sensitive to heat stress and the expression of some of these small RNAs was affected^22,24,25^. Using RNA-seq, comprehensive analyses of heat treatments in NHCC identified 9,687 novel lncRNAs under heat stress in *B*. *rapa*^21^. Moreover, due to promoter demethylation, high *BramMDH1* expression was exhibited in cold-acclimated *B*. *rapa* and the overexpression of *BramMDH1* enhanced heat-tolerance and the growth rate in *Arabidopsis*^26^. Although some studies on the role of lncRNAs in response to abiotic stress have been performed, few studies have reported comprehensive surveys of lncRNAs in heat tolerance of non-heading Chinese cabbage.

In many plants, numerous genes responsive to heat stress have been found using genomic, transcriptomic, and proteomic analyses^27,28^. However, how long non-coding RNAs regulate gene expression together with miRNAs modulating transcriptome responses to heat stress in *B*. *rapa* is still ambiguous. In this study, we systematically identified and characterized the lncRNAs in *B*. *rapa* under heat stress at a genome-wide scale using strand-specific RNA-seq (ssRNA-seq). Integrating mRNA and miRNA expression patterns, here we shed light on the landscape of non-coding RNAs for heat tolerance and reveal their regulatory roles in non-heading Chinese cabbage.

## Results

### Identification and characterization of lncRNAs in Chinese cabbage

**Figure 1:**
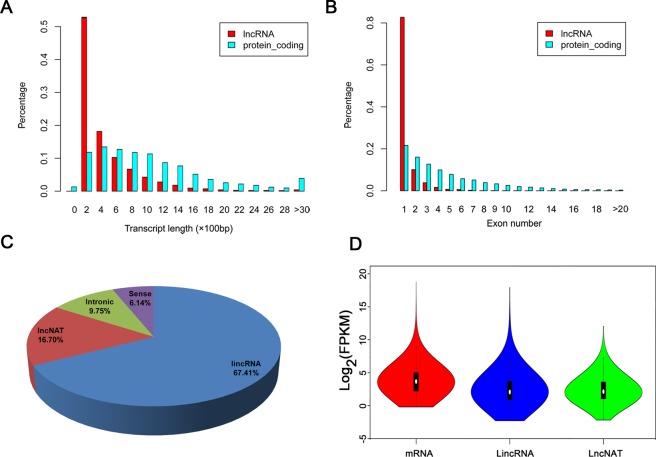
Characteristics of lncRNAs in Chinese cabbage. (**A**) The proportions of transcript lengths in lncRNAs and protein-coding genes. (**B**) The distribution of exon numbers per transcript for lncRNAs and protein-coding genes. (**C**) Composition of different types of lncRNAs. (**D**) The expression levels of different types of lncRNAs (fragments per kilobase per million, FPKM).

The systematic genome-wide identification of lncRNAs in Chinese cabbage was performed using strand-specific RNA-seq of leaves at three different stages (0 h, 1 h and 12 h) under heat stress. The sequencing data from the libraries showed highly correlation relationship in the repeats at each stage in the two varieties (Fig. S1). A total of 128 million clean reads were generated using the Illumina HiSeq. 2500 platform (Table S1). The genome sequence of *B*. *rapa* (Chiifu-401-42) retrieved from the Brassica Database (BRAD) (http://brassicadb.org/brad/) was used as the reference database^29^. Overall, 4941 lncRNAs were identified from two varieties and 4594 of them belong to novel lncRNAs (Fig. 1 and Table S2). These novel lncRNAs were distributed on all the chromosomes with the largest number of lncRNAs on the chromosome A09 (Table S2). Two of the lncRNAs (TCONS_00005147 and TCONS_00053016) showed similar with previous reported lncRNAs (AT4 or IPS1 and MSUR1) in *Arabidopsis* and *Mus musculus*, respectively (http://www.lncrnadb.org). Further analysis of the homology of lncRNAs in the three *Brassica* species (*B*. *rapa*, *B*. *oleracea* and *B*. *napus*) found that 3.78% (187/4941) of these lncRNAs were located in the same regions as previous report and 29.41% and 10.70% of the 187 lncRNAs showed homology with *B*. *napus* and *B*. *oleracea*, resepectively (Table S3)^30^. We also analyzed the sequence conservation of lncRNAs in *Arabidopsis* and rice with E < 1e-20 using BLASTn algorithm. Around 2.48% (114/4594) and 0.04% (2/4594) showed sequence conservation with *Arabidopsis* and rice, respectively.

We then characterize the basic genomic features of these lncRNAs. Compared with the protein-coding genes, the lncRNAs were mainly 200–1200 bp in length with fewer exons, as most of them contained a single exon (Fig. 1A,B). The results suggested that the majority of the *B*. *rapa* lncRNAs have shorter lengths and fewer exons than those of protein-coding genes. According to their genomic locations, these lncRNAs were classified into 3097 lincRNAs (67.41%), 767 lncNAT (16.70%), 282 sense lncRNAs (6.14%), 448 intronic lncRNAs (9.75%) (Fig. 1C). Using FPKM (fragments per kilobase per million fragments mapped), the expression levels of the lncRNAs and protein coding genes were calculated and compared at different stages. The expression levels of intergenic lncRNAs (lincRNAs) and antisense lncRNAs (lncNAT) was lower than those of mRNAs (Fig. 1D).

### Differential expression and the target genes of lncRNAs

**Figure 2:**
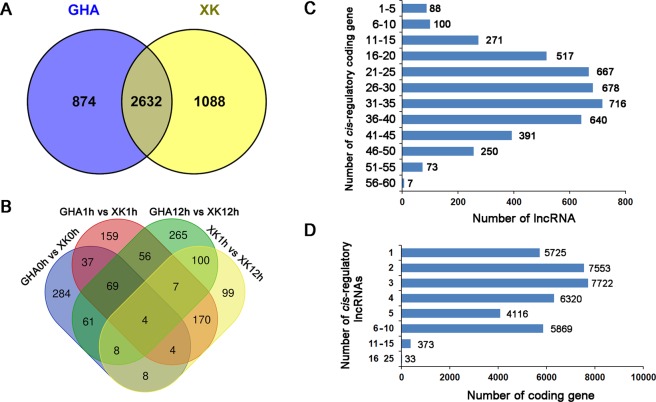
Differential expression of lncRNAs and *cis*-regulated protein coding genes by lncRNAs. (**A**) Venn diagram showing the shared and distinct lncRNA expression in the heat sensitive variety ‘GHA’ and tolerant variety ‘XK’. (**B**) Venn diagram showing the overlap of differentially expressed lncRNAs among different comparisons. (**C**) The number of target protein-coding genes regulated by lncRNA. (**D**) The number of lncRNAs that have potential *cis*-regulatory effects on protein-coding genes.

Totally, 3506 and 3720 lncRNAs were expressed in ‘GHA’ and ‘XK’, respectively, with 2632 shared in both varieties (Fig. 2A). A total of 1686 lncRNAs were differentially expressed between the heat sensitive variety ‘GHA’ and heat tolerant variety ‘XK’ at the three stages under heat stress (Table S4). Comparison of the six samples revealed that only 4 differentially expressed lncRNAs were shared at all stages (Fig. 2B). And there were 69 shared lncRNAs between ‘GHA’ and ‘XK’ at the three stages (0 h, 1 h and 12 h).

LncRNAs have been found to regulate the expression of proximal and distal protein-coding genes through *cis*- (regulation of neighboring loci) and *trans*-acting modes^31^. The proximal protein-coding genes located within a genomic window of 100 kb on the same chromosome were searched as *cis*-regulated target genes of lncRNAs. A total of 4390 lncRNAs were found to have potential *cis*-regulatory effects on 37,533 protein-coding genes (Table S5). Among them, more than 90% regulate ten to fifty target genes, and 80 lncRNAs have up to 50 target genes (Fig. 2C). Most of the protein-coding genes corresponded to one to ten lncRNAs, and 406 protein-coding genes were *cis*-regulated by up to ten lncRNAs (Fig. 2D). In addition, a total of 753 lncRNAs and 846 associated target protein-coding genes were determined to be *trans*-regulated in 1005 gene pairs under heat stress (Table S5).

### Identification of heat responsive lncRNAs and genes

**Figure 3:**
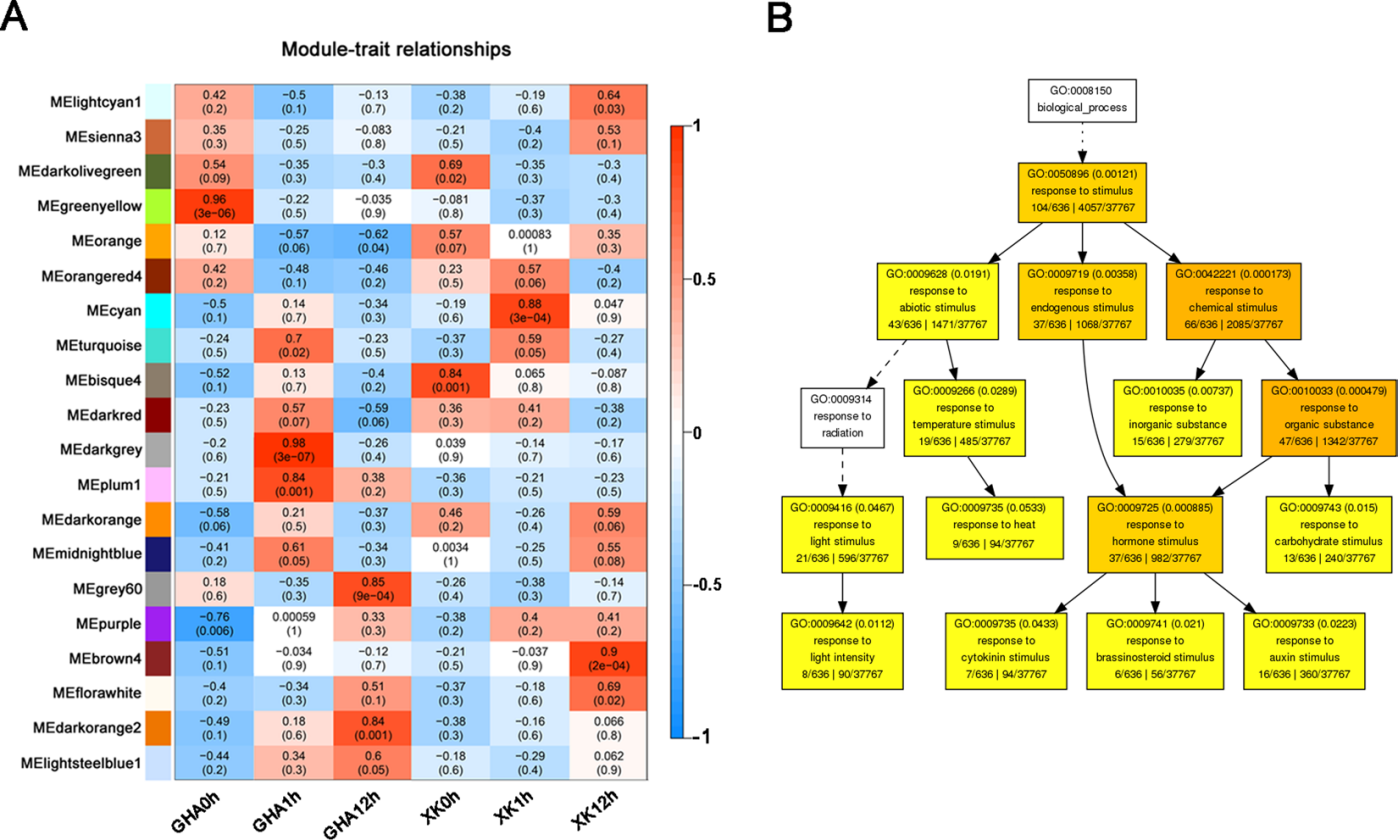
Weighted gene co-expression network analysis (WGCNA) and GO enrichment of differentially expressed genes (DEGs). (**A**) Module-heat response correlations within 20 module eigengenes. The table is color-coded by correlation between the modules and traits. Each row corresponds to a module eigengene and each cell contains the corresponding correlation value and *P*-value. (**B**) GO enrichment analysis of the DEGs in the module ‘MEcyan’. The genes in module ‘MEcyan’ is significantly correlated with the tolerant variety ‘XK’ at 1 hour (XK1h) after heat stress (See Fig. 3A).

To further examine the regulation of lncRNAs on protein-coding genes, the co-expression relationship between them was analyzed by a weighted gene co-expression network analysis (WGCNA). The co-expression network was divided into 20 modules with different gene expression patterns for each module (Figs 3A and S2). Module-trait relationships revealed that six modules (MEgreenyellow, MEdarkgrey, MEgrey60, MEbisque4, MEcyan and MEbrown4) were significantly correlated with each stage (Fig. 3A). GO enrichment analysis also showed that most of these genes were involved in “response to stimulus”, “response to biotic stimulus” and “response to hormone stimulus” (Fig. 3B, Tables 1 and S6). Particularly, the DEGs of the module MEcyan at 1 h after heat stress were enriched in ‘response to stimulus’, including light stimulus, heat, hormone and carbohydrate stimulus (Fig. 3B). These results suggested that dozens of genes were differentially expressed in response to heat stress at the early stage (1 h).

**Table 1:** GO analysis (biological process) of lncRNAs and regulated protein coding genes in the competing endogenous RNA (ceRNA) network under heat stress.

GO ID	Description	*P* value	FDR
GO:0043401	steroid hormone mediated signaling pathway	3.00E-07	6.40E-05
GO:0009741	brassinosteroid mediated signaling pathway	3.00E-07	6.40E-05
GO:0050896	response to stimulus	4.10E-06	0.00052
GO:0006351	transcription, DNA-templated	7.20E-06	0.00074
GO:0042221	response to chemical	1.10E-05	0.00085
GO:0065007	biological regulation	1.50E-05	0.0011
GO:0007165	signal transduction	1.90E-05	0.0013
GO:0009266	response to temperature stimulus	2.40E-05	0.0014
GO:0009408	response to heat	2.40E-05	0.0014
GO:0009719	response to endogenous stimulus	5.20E-05	0.0023

**Figure 4:**
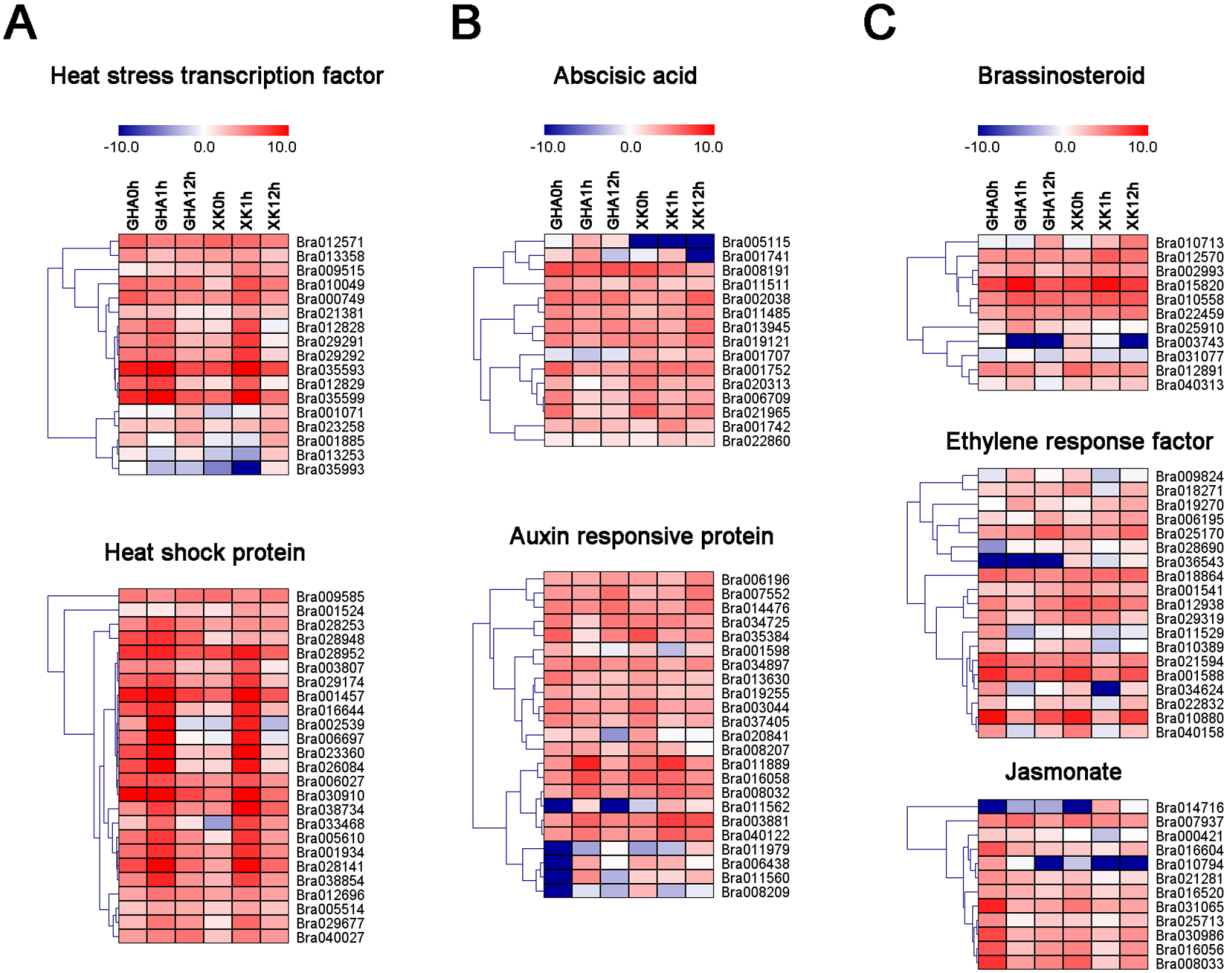
Heatmaps of the differentially expressed genes (DEGs) involved in phytohormone pathways and transcription factors at different stages under heat stress. All the heatmaps were generated using MeV4.9 software with log_2_ transformed FPKM values. (**A**) Heat stress transcription factors (HSFs) and heat shock proteins (HSPs). (**B**) DEGs involved in abscisic acid (ABA) and auxin pathways. (**C**) DEGs involved in brassinosteroid (BR), ethylene and jasmonate pathways.

**Figure 5:**
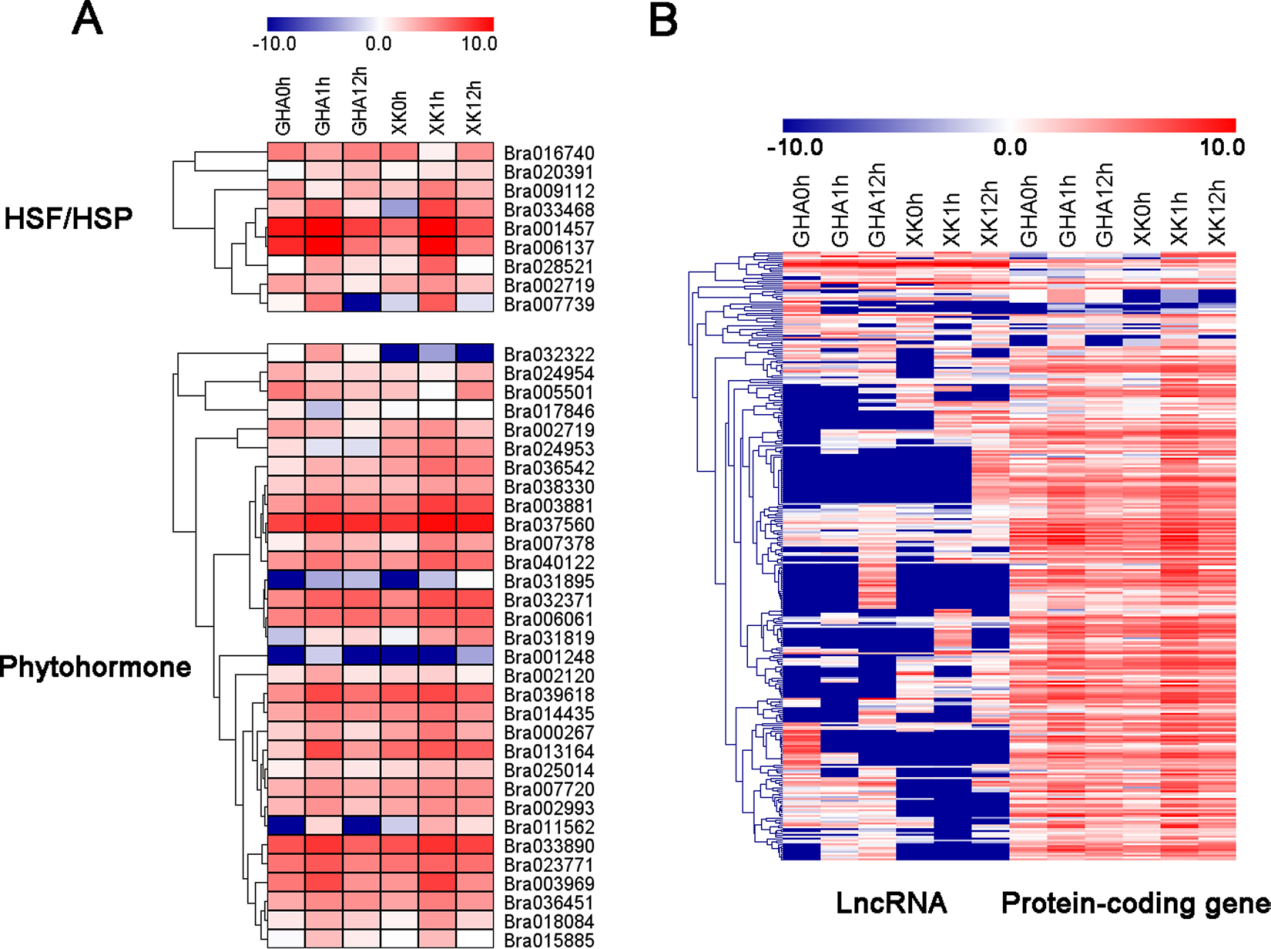
Heatmaps of the differentially expressed genes (DEGs) involved in “response to stimulus” and the expression patterns of associated lncRNAs. All the heatmaps were generated using MeV4.9 software with log2 transformed FPKM values. (**A**) The expression patterns of transcription factors (HSF/HSP) and DEGs involved in phytohormone pathways. (**B**) Heatmaps showing the expression patterns of lncRNAs involved in “response to stimulus” and their *cis*-regulated genes.

The expression patterns also demonstrated that HSF, HSP and the genes related to plant hormones (auxin, ethylene, and brassinosteroid) showed changes in their expression levels (Fig. 4 and Table S7). In total, 17 HSFs and 24 HSPs were differentially expressed during heat stress and most of them up-regulated at 1 h after heat treatment (Fig. 4A and Table S7). A total of 85 DEGs involved in ‘response to hormone stimulus’ were identified, including auxin, BR, GA, jasmonic acid (JA), SA and ABA pathways (Fig. 4B,C and Table S7). We identified 23 and 11 DEGs that participated in auxin and BR pathways, respectively (Table S7). Most of the DEGs involved in auxin pathway were induced in the heat tolerance variety ‘XK’ (Fig. 4B). Particularly, two BR signaling proteins BZS1 (Bra010713) and BZR1 (Bra012570) were up-regulated at 1 h after heat treatment (Fig. 4C). In addition, other transcription factors (TFs), such as Bra009112 (DREB2A), were also induced at 1 h after heat treatment (Fig. 5A). Some of these TFs and phytohormone related DEGs were *cis*-regulated by the 323 lncRNAs which were involved in ‘response to stimulus’ (Fig. 5B, Tables S5 and S7). Thus, these protein-coding genes and lncRNAs were co-expressed under heat stress.

**Figure 6:**
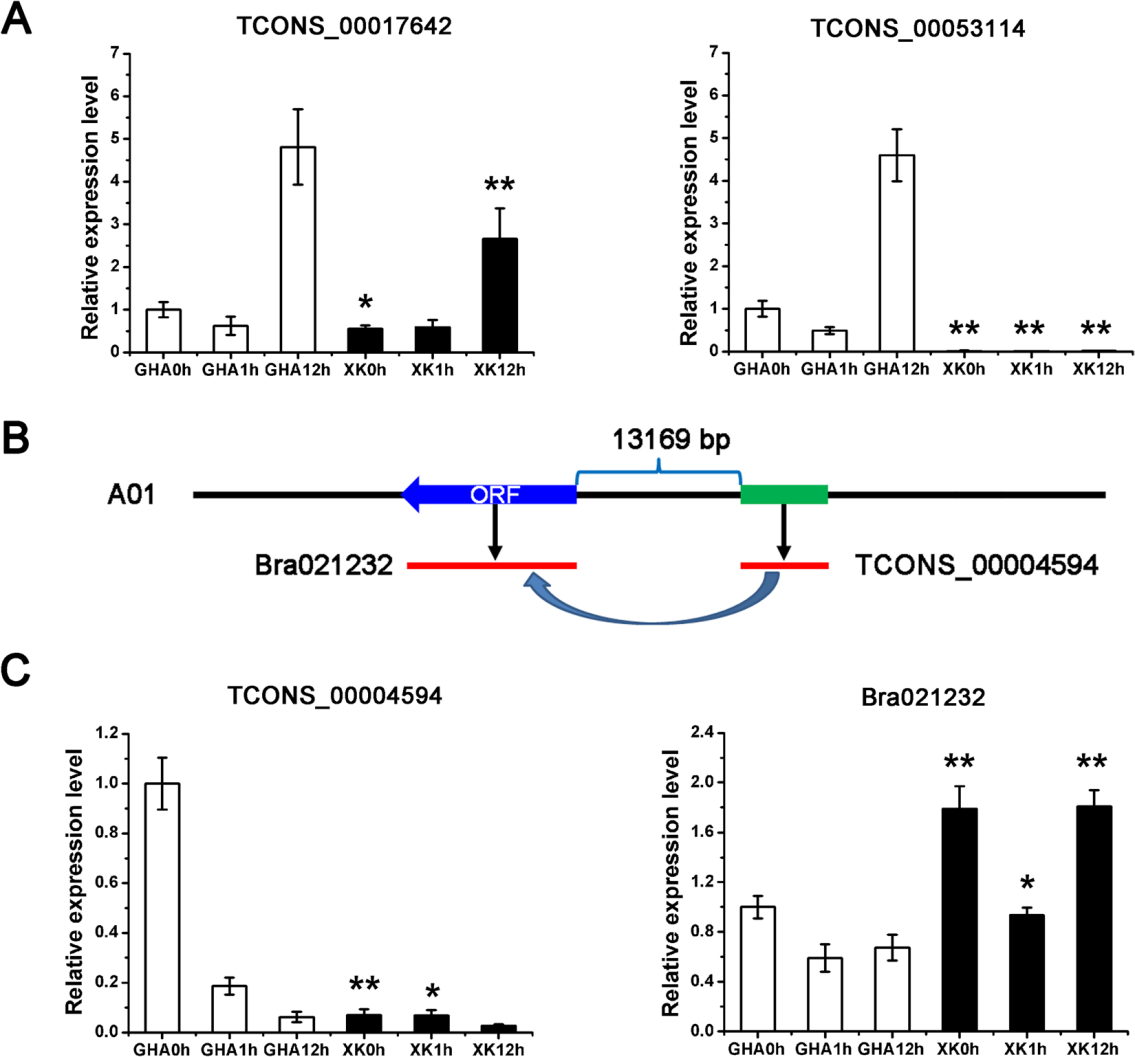
Quantitative RT-PCR (qRT-PCR) validation of differentially expressed lncRNAs and the *cis*-regulated protein genes. (**A**) Quantitative RT-PCR (qRT-PCR) validation of two lncRNAs (TCONS_00017642 and TCONS_00053114). (**B**) Gene structures of the lncRNA (TCONS_00004594) and nearby protein-coding gene (Bra021232). (**C**) qRT-PCR validation of the lncRNA (TCONS_00004594) with its target gene (Bra021232). Significant differences of the expression level between the sensitive variety ‘GHA’ and tolerant variety ‘XK’ at different stages were determined by Student’s *t*-rest (*P < 0.05 or **P < 0.01).

There were 506 differentially expressed lncRNAs at 1 h after heat treatment, including 355 up-regulated and 151 down-regulated lncRNAs in the heat tolerance variety ‘XK’ (Fig. 2B and Table S7), respectively. The results indicated that more responsive lncRNAs were induced at the early stage under heat stress. Three differentially expressed lncRNAs (TCONS_00017642, TCONS_00053114 and TCONS_00004594) were validated by qRT-PCR (Fig. 6). Furthermore, the lncRNA TCONS_00004594, which is located downstream at the protein-coding gene Bra021232, *cis*-regulated its expression level (Fig. 6B). Quantitative RT-PCR confirmed the negative association of expression levels of TCONS_00004594 and Bra021232 (Fig. 6C).

### LncRNAs interact with miRNAs as potential targets or target mimics

**Figure 7:**
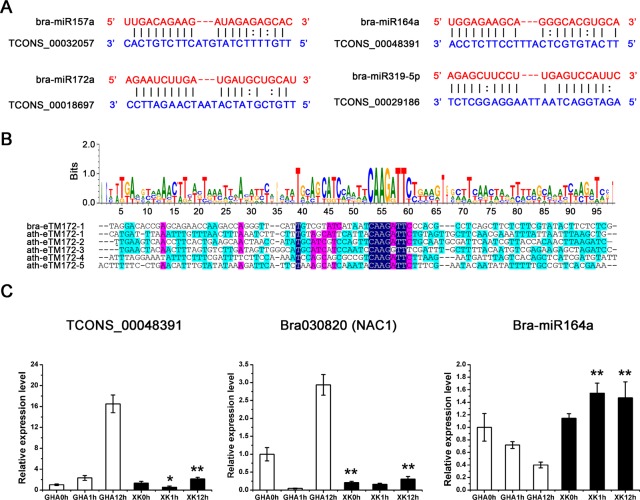
LncRNA acting as endogenous target mimics (eTMs) of miRNAs in Chinese cabbage and quantitative RT-PCR (qRT-PCR) validation of bra-miR164a with its target. (**A**) Four lncRNAs were identified as eTMs of miRNAs. The blue sequences are *B*. *rapa* lncRNAs and the red sequences indicate the regions which complemented by miRNAs. (**B**) Sequence alignment of eTMs for miR164a (eTM164s) in *B*. *rapa* and *Arabidopsis thaliana*. The bases in the colored background are the sequences paired to miR164. (**C**) qRT-PCR validation of bra-miR164a with its target gene (Bra030820) and eTM (TCONS_ 00048391). Significant differences of the expression level between the sensitive variety ‘GHA’ and tolerant variety ‘XK’ at different stages were determined by Student’s *t*-rest (*P < 0.05 or **P < 0.01).

LncRNAs and miRNAs are both non-coding RNAs and their interactions play a role in the regulation of development in many plants^32^. Previous studies have reported that lncRNAs can be targeted by miRNAs and could also function as endogenous target mimicry (eTM) as well as potential miRNA precursors^16,33^. Some lncRNAs may act as competing endogenous RNAs (ceRNAs) in the regulation of gene expression^34^. In our study, we identified 67 lncRNAs as potential targets of 55 miRNAs and 40 lncRNAs as eTMs of 36 miRNAs (Tables S8 and S9). Interestingly, 23 miRNAs (including bra-miR156a-5p, bra-miR157a and bra-miR164a), not only regulated lncRNAs but also were targeted by eTMs (Tables 2, S8 and S9). For example, bra-miR164a regulates two lncRNAs (TCONS_00007624 and TCONS_00030024) and NAC TFs through degradation at post-transcriptional level (Table 2). However, two other lncRNAs (TCONS_00048391 and TCONS_00010856) regulate bra-miR164a as target mimics and inhibit its function (Fig. 7A and Table 2). In addition, 46 lncRNAs were predicted to be precursors of miRNAs in Chinese cabbage in our study (Table S10).

**Table 2:** Some lncRNAs were predicted as targets or endogenous target mimics (eTMs) of miRNAs in Chinese cabbage.

LncRNA	miRNA	miRNA sequence	LncRNA sequence
**Target**			
TCONS_00042803	bra-miR156a-5p	UGACAGAAGAGAGUGAGCAC	UGACAGAAGAGAGUGAGCAC
TCONS_00032634	bra-miR157a	UUGACAGAAGAUAGAGAGCAC	UUGACAGAAGAUAGAGAGCAC
TCONS_00036147	bra-miR158-5p	CUUUGUCUAUCGUUUGGAAAAG	CUUUGUCUAUCGUUUGGAAAAG
TCONS_00035474	bra-miR159a	UUUGGAUUGAAGGGAGCUCUA	UUUGGAUUGAAGGGAGCUCUA
TCONS_00023440	bra-miR160a-5p	UGCCUGGCUCCCUGUAUGCCA	UGCCUGGCUCCCUGUAUGCCA
TCONS_00023440	bra-miR160a-3p	GCGUAUGAGGAGCCAUGCAUA	GCGUAUGAGGAGCCAUGCAUA
TCONS_00012562	bra-miR162-5p	GGAGGCAGCGGUUCAUCGAUC	GGAGGCAGCGGUUCAUCGAUC
TCONS_00007624	bra-miR164a	UGGAGAAGCAGGGCACGUGCA	UGGAGAAGCAGGGCACGUGCA
TCONS_00030024	bra-miR164a	UGGAGAAGCAGGGCACGUGCA	UGGAGAAGCAGGGCACGUGC
TCONS_00024317	bra-miR167a	UGAAGCUGCCAGCAUGAUCUA	UGAAGCUGCCAGCAUGAUCUA
TCONS_00000650	bra-miR168a-5p	UCGCUUGGUGCAGGUCGGGA	UCGCUUGGUGCAGGUCGGGA
TCONS_00037699	bra-miR171a	UUGAGCCGUGCCAAUAUCACG	UUGAGCCGUGCCAAUAUCACG
TCONS_00049086	bra-miR172a	AGAAUCUUGAUGAUGCUGCAU	AGAAUCUUGAUGAUGCUGCAU
TCONS_00014220	bra-miR391-5p	UUCGCAGGAGAGAUAGCGCCA	UUCGCAGGAGAGAUAGCGCCA
TCONS_00038487	bra-miR396-5p	GCUCAAGAAAGCUGUGGGAAA	GCUCAAGAAAGCUGUGGGAAA
**eTM**			
TCONS_00013120	bra-miR156a-5p	UGACAGAAGA---GAGUGAGCAC	GGCUUCACUCUUCUCUUCUGACA
TCONS_00032057	bra-miR157a	UUGACAGAAG---AUAGAGAGCAC	UUGUUUUCUAUGUACUUCUGUCAC
TCONS_00052940	bra-miR159a	UUUGGAUUGA---AGGGAGCUCUA	AACGGUGCCCUUUGUCGAUCCAAA
TCONS_00033140	bra-miR160a-3p	GCGUAUGAGG---AGCCAUGCAUA	UGUGAAGGGUUCCUCUUCAUACGC
TCONS_00026882	bra-miR162-5p	GGAGGCAGCG---GUUCAUCGAUC	GCUUGAUGACGCUACGCUGCCUCC
TCONS_00048391	bra-miR164a	UGGAGAAGCA---GGGCACGUGCA	UUCAUGUGCUCAUUUCCUUCUCCA
TCONS_00010856	bra-miR164a	UGGAGAAGCA---GGGCACGUGCA	AUCGUUUGCCCGGAUGCUUCUUCA
TCONS_00018697	bra-miR172a	AGAAUCUUGA---UGAUGCUGCAU	UUGUCGUAUCAUAAUCAAGAUUCC
TCONS_00033849	bra-miR390-5p	AAGCUCAGGA---GGGAUAGCGCC	UGCAGUGUUUCCCAUCUUGAGCUU
TCONS_00038455	bra-miR408-5p	GGGAGCCAGG---GAAGAGGCAGU	GAGCUCUCUUCAUCUCGGGCUCUC

The miRNA target mimicry is similar to the interactions of miRNAs and their targets, and eTMs can also rely on sequence-dependent interactions with lncRNAs^16^. To investigate the evolutionary conservation of eTMs, we aligned the sequences of the predicted eTM binding sites of bra-miR172a in *B*. *rapa* and *A*. *thaliana*^33^. The results showed that miRNA binding sites were well conserved among eTMs of the same miRNA in the two species, while the nucleotides forming the bulge region in eTMs were not conserved (Fig. 7B). This finding is consistent with the results from floral buds of *B*. *rapa*^20^. Furthermore, we analyzed the expression level of bra-miRNA164a and its target or eTM to understand the relationship between miRNA target mimics and related miRNAs. We found that bra-miR164a was significantly up-regulated in the heat tolerance variety ‘XK’, while the eTM (TCONS_00048391) and the target gene (Bra030820, NAC1) were down-regulated at the same stages under heat stress (Fig. 7C), indicating the competing binding for bra-miR164a.

### LncRNA-miRNA-mRNA co-expression networks under heat stress

**Figure 8:**
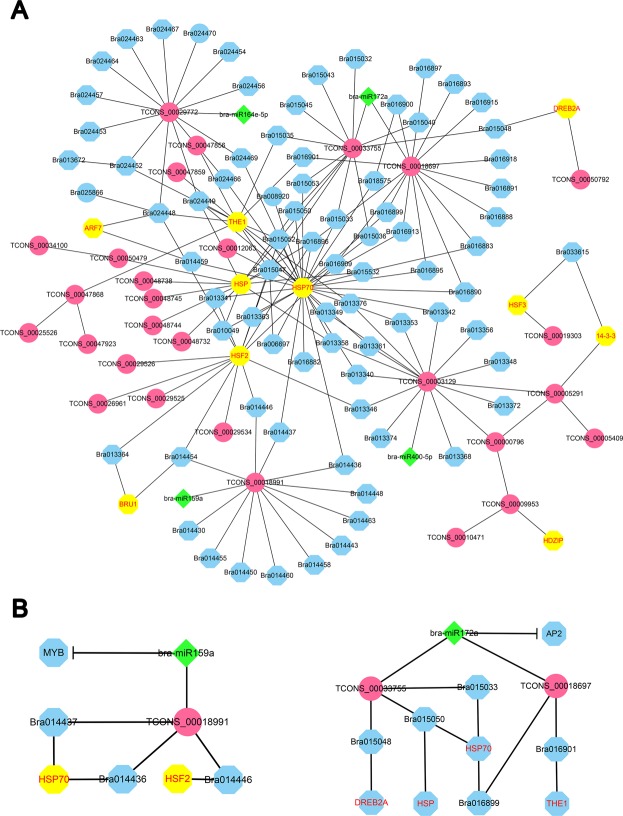
Co-expression network of lncRNA-miRNA-mRNA. (**A**) Network of lncRNA with *cis*-regulated protein coding genes as well as four miRNAs (bra-miR164e-5p, bra-miR172a, bra-miR159a and bra-miR400-5p), including some known heat responsive genes (HSP, HSF and DREB2A). Red circle, lncRNAs; green triangle, miRNAs; blue octagon, mRNAs. Yellow octagon with red font, known heat responsive genes. (**B**) Two examples of sub-networks containing bra-miR159a and bra-miR172a, respectively. Bra-miR159a and bra-miR172a negatively regulated their target genes MYB and AP2, respectively.

It has been reported that lncRNAs can function as endogenous miRNA sponges^33^. Additionally, lncRNAs and mRNAs might interact through competition with the same miRNA, or miRNAs and lncRNAs could interact with each other, thus regulating the function of the same mRNAs^35,36^. Integrated analysis of target prediction and functional enrichment based on co-expression networks of lncRNA-miRNA-mRNA may provide a clue for comprehensively understanding the regulation of ncRNAs in plants. In this study, we constructed a co-expression network in Chinese cabbage under heat stress, including 210 DEGs, 4 miRNAs and 33 lncRNAs (Fig. 8A). GO enrichment analysis showed that the biological functions of these ncRNAs were involved in “brassinosteroid mediated signaling pathway”, “response to stimulus”, “response to heat” and so on (Table 1), suggesting that they might contribute to the heat response through these signaling pathways. The results showed a clear heat tolerance co-expression network and six lncRNAs (TCONS_00029772, TCONS_00033755, TCONS_00018697, TCONS_00018991, TCONS_00003129 and TCONS_00005291) were identified as key lncRNAs (Fig. 8A). These key lncRNAs can *cis*-regulated the protein coding genes or through its miRNA response elements (MREs) to compete for miRNA and to regulate the expression of the target genes. For instance, the competition of lncRNAs and protein coding genes for binding to the miRNAs (bra-miR159a or bra-miR172a) may led to the regulation of the target genes or heat-responsive genes (HSPs, HSFs, THE and DREB2A) by lncRNAs (Fig. 8B).

## Discussion

As consequence of the greenhouse effect, increasing temperatures constraint plant growth, development, and productivity. Thus, plants should evolve special adaptive mechanisms to cope with heat stress^9^. Understanding the mechanism of gene regulation, especially the non-coding RNAs (ncRNAs), will provide a molecular basis for better adaptation to heat stress in non-heading Chinese cabbage. In *B*. *rapa*, systematic analysis of lncRNAs during pollen development and fertilization has identified 12,051 putative lncRNAs^20^. And the comprehensive analysis of heat treatments in NHCC using RNA-seq also identified 9,687 novel lncRNAs in Chinese cabbage^21^. However, few studies have been reported about heat tolerance associated lncRNAs and the co-expression network of lncRNA-miRNA-mRNA in the regulation during heat stress in non-heading Chinese cabbage. In this study, using strand-specific RNA-seq, a total of 4594 novel lncRNAs were systematically identified to the early heat responsive stage in Chinese cabbage. Co-expression networks were also constructed to predict the functions of lncRNAs involved in high temperature responses.

Compared with mRNAs, lncRNAs show little conservation at the primary sequences and some lncRNAs show striking conservation at the level of synteny and function^37^. In the present study, we also found that lncRNAs of *B*. *rapa* had relatively high sequence conservation in *B*. *napus* and *B*. *oleracea* at the genus level (Table S3)^38^. However, highly divergence in the sequences were observed, when compared with other genus plants, such as *Arabidopsis* or rice. Interestingly, in the lncRNAs that harbored miRNA binding sites, the sequence of the motif was conserved at the same locus in all Brassicaceae (Fig. 7B), indicating that they may be involved the similar biological process and conserved lincRNA regulatory pathway^39,40^. These sequence homology or position conservation of lncRNAs may give some insights into the evolutionary dynamics of them and conservation of function in plants^38,39^.

LncRNAs can regulate gene expression either in *cis* or in *trans* and some of them are overlapped^31,41^. In our study, we predicted 37,533 *cis* and 906 *trans* target genes, and 785 were overlapped, indicating the complexity of the roles of lncRNAs under heat stress (Table S5). Many *cis*-acting lncRNAs have been demonstrated to activate the expression of flanking genes^20^. Thus, we performed a co-expression analysis among the differentially expressed lncRNAs with their targeted genes and related miRNAs (Fig. 8). GO enrichment analysis showed that these ncRNAs and mRNAs were involved in “brassinosteroid mediated signaling pathway”, “response to stimulus”, “response to heat” and so on (Table 1).

Plant hormones play important roles in regulating responses to biotic and abiotic stresses^42^. In *Brassica juncea*, phytohormones were induced to ameliorate heat stress, and soaking seeds in 100 μM IAA, 100 μM GA, and 0.5 μM ABA were effective for mitigating the effect of heat stress^43^. Although auxins known to be associated with plant development, several recent studies have highlighted their roles in response to abiotic stress^42^. In developing anthers of plants, endogenous auxin is reduced in response to heat stress^44^. In this study, some auxin responsive genes, including Bra003881 and Bra040122, were induced in the heat tolerance variety ‘XK’ (Fig. 4B and Table S7). Salicylic acid is also found to be an effective protectant under heat stress. In grape, the effects of SA were thought to be associated with enhancing the expression level of heat shock protein 21 (HSP21)^45^. In our study, we also found that two DEGs (Bra004787 and Bra005168) were induced by SA at 1 h or 12 h after heat treatment (Table S7). We also found that 19 ERF and 12 JA genes were differentially expressed under heat stress (Fig. 4C and Table S7). Studies on the *cpr5-1 npr1-1* double mutant of *Arabidopsis*, indicated that SA plays a role in basal thermotolerance, which may be contributed by JA or ethylene^46^.

Under heat stress, the lncRNA (TCONS_00016454) was down-regulated in heat tolerant variety ‘XK’, while its target BES1/BZR1 homolog 3 (Bra012570) was responsive to high temperatures (Tables S4 and S7). When mustard (*B*. *juncea*) seedlings were treated with different concentrations of 24-epibrassinolide (24-EBL), they showed better growth and enhanced protein content under heat stress as well as activated the antioxidant enzymes^47^. EBL-induced thermotolerance is correlated with the expression of major classes of HSPs and the enhanced protection of the translational machinery from degradation following heat stress^8,48^. HSP90 has been reported to be involved in BR signaling via interactions with BES1/BZR1, regulating a set of BR responsive genes^49^.

Target mimicry has recently been reported to be an important function of lncRNAs in plants^50^. *Arabidopsis* IPS1 is the first target mimic identified to regulate the expression of miR399^51^. In this study, we predicted 40 potential eTMs for 36 miRNAs in Chinese cabbage. Some of these eTMs were differentially expressed in Chinese cabbage under heat stress, including TCONS_00013120 (bra-miR156a-5p) and TCONS_00038455 (bra-miR408-5p) (Tables 2 and S4). The eTM binding sites for the same miRNAs were found to be well conserved in *B*. *rapa* and *A*. *thaliana* (Fig. 7B). Thus, we propose that certain interactions between these potential eTMs and miRNAs may exist and play a fundamental role in heat tolerance. Heat responsive lncRNA (TCONS_00048391) is an eTM of bra-miR164a, and could be a sponge for miRNA binding, reducing the expression of bra-miR164a (Fig. 7). This lncRNA may be a ceRNA for the target gene (NAC1, Bra030820) of miR164a in Chinese cabbage (Fig. 7C). In *Arabidopsis*, overexpression of the ANAC042 has been reported to increase tolerance to heat stress^52^. These results suggested that this network might be involved in the lncRNA (TCONS_00048391) regulation of heat tolerance through miR164a and *NAC* gene in non-heading Chinese cabbage.

It has been documented that lncRNA can act as a decoy for miRNA binding, forming a ceRNA network by regulating the abundance of the target transcripts of the same miRNAs^35^. In the present study, based on the *cis*-regulation of lncRNAs and miRNA-target interactions, we constructed a co-expression network comprising lncRNAs, miRNAs and miRNAs (Fig. 8). Four miRNAs (bra-miR159a, bra-miR164e-5p, bra-miR172a and bra-miR400-5p) were included in the network and involved in the regulation of heat responsive genes (HSP70, DREB2A and THE1). In wheat, the overexpression of miR159 led to the down regulation of target GAMYB and to decrease plant heat tolerance^14^. These results showed that miRNAs occupied a central position by supplying intermediate bridges for lncRNAs and mRNAs and indicated that these four miRNAs were involved in heat tolerance in Chinese cabbage.

Heat stress factors (Hsfs) and heat shock proteins (HSPs) play a central role in heat stress and acquired thermotolerance in plants. HSPs are molecular chaperones, which protect proteins against irreversible heat-induced damage by assisting in the folding and sorting of newly synthesized polypeptides^53–55^. In *Arabidopsis* and carnation, the expression of HsfA3 in response to heat stress, has been shown to be dependent on the transcription factor DREB2A (dehydration-responsive element binding protein 2A)^53^. In the co-expression network, we also found a DREB2A gene (Bra009112) to be taken part in the regulation subnetwork of miR172a-lncRNA (TCONS_00033755) interaction (Fig. 8C). In *Arabidopsis*, DREB2A has a negative regulatory domain in the central region and the deletion of this region transforms DREB2A into a constitutively active form (DREB2A CA). The overexpression of DREB2A CA in transgenic *Arabidopsis* enhanced tolerance to heat stress^56^. Thus, the bra-miR172a-lncRNA interaction regulated the target genes associated with heat tolerance in Chinese cabbage.

Taken together, in this study, we constructed co-expression networks among a series of miRNAs, mRNAs, and lncRNAs in Chinese cabbage under heat stress. Further investigation of these lncRNAs that are highly co-expressed with coding target genes of known functions may elucidate the gene expression regulation pathway involving lncRNAs in the heat tolerance of Chinese cabbage.

## Materials and Methods

### Plant materials

A heat sensitive variety “GHA” and a tolerant variety “XK” of non-heading Chinese cabbage (*B*. *rapa* ssp. chinensis) were used in this study. Sterile seeds were sown in pots and germinated in a growth chamber. For heat treatments, 3-week-old seedlings were grown at 37 °C (high temperature) for 1 and 12 h. For control samples (CK), the leaf discs were incubated at 25 °C. Then, the leaves of the two varieties under CK and heat treatments were collected, frozen immediately in liquid nitrogen, and stored at −80 °C for use.

### RNA extraction, library construction and Illumina sequencing

Total RNA was isolated from the leaves at different stages using Trizol Reagent (Invitrogen, USA) according to the manufacturer’s instructions. The extracted RNA was treated with DNase I (Promega, USA) to remove the contaminated DNA. The RNA quality and integrity were verified by using the Nanodrop 2000 and Agilent Bioanalyzer 2100 System (Agilent Technologies, USA).

For ssRNA-seq library construction, 5 μg of total RNA from each sample was used for rRNA removal using the Ribo-Zero rRNA Removal Kit (Epicentre, Madison, USA). Sequencing libraries were generated using the NEBNext Ultra^TM^ Directional RNA Library Prep Kit according to the manufacturer’s instructions. Briefly, using random hexamer primers, the cDNA was synthesized by reverse transcriptase, DNA polymerase I and RNase H. After adenylation of the 3’ ends of DNA fragments, NEBNext Adaptor was ligated to prepare for hybridization. The library fragments were then purified with AMPure XP Beads (Beckman Coulter, USA) to select insert fragments of 150~200 bp. The quality and quantity of the libraries were verified using an Agilent 2100 Bioanalyzer and ABI real time RT-PCR, respectively. Then, the libraries were purified and sequenced on an Illumina Hiseq. 2500 platform with a 100 bp paired-end. Two biological replicates were performed for each sample. All the clean reads were deposited in the NCBI Sequence Read Archive under the accession: SRP187925.

### Genome-wide identification of lncRNA

The *B*. *rapa* reference genome and gene annotations were downloaded from the Brassica Database (BRAD) (http://brassicadb.org/brad/)^29^. The raw reads were filtered by removing adaptors and trimming low-quality reads. The clean reads of each sample were mapped onto *B*. *rapa* reference genome using TopHat 2.0 program^57^. After the alignment, Cufflinks 2.0 was used to assemble reads into transcripts and merged to generate final transcripts. The fragments per kilobase of transcript per million mapped reads (FPKM) of the transcripts were calculated. Transcripts that overlapped with known protein-coding genes, transcripts with FPKM scores <0.5 and the length shorter than 200 nucleotides were discarded. Then, the CPC (Coding Potential Calculator) and CNCI (Coding Non Coding Index) programs were used to predict the coding potential of the remaining transcripts^58,59^. All the transcripts with CPC scores >0 or CNCI >0 were removed. Finally, the retained transcripts were candidate lncRNAs. The genome sequences of the lncRNAs of *Arabidopsis* and rice were downloaded from the Green Non-Coding Database (GREENC) (v1.12)^60^ for sequence conservation analysis.

### Analysis of differential expression patterns and functional annotations

Quantification of the expression level of protein-coding transcripts and candidate lncRNAs were calculated as FPKM using Cufflinks package^61^. Differential expression was determined using the DESeq with |log2(fold change)| ≥ 1 and an FDR (false discovery rate) threshold of 5%^62^. The K-means clustering of the differentially expressed transcripts was analyzed by Microarray Experiment Viewer (MeV, v4.9) software^63^ basing on their log_2_ values of transcript abundances with default parameters. Functional annotations of the DEGs and candidate lncRNAs were performed to search against the Gene Ontology (GO) and KEGG databases^64^. GO enrichment and metabolic pathway analyses were carried out using ArigGO^65^ with default parameters. Over-representing GO categories were detected using a Fisher’s exact test and Honchberg adjusted *p*-value < 0.05.

### Target gene and target mimics prediction of lncRNAs

LncRNAs were predicted via two independent algorithms: *cis*- or *trans*-regulatory effects^66^. These genes transcribed within a 100 kb window upstream or downstream of lncRNA were defined as potential *cis* target genes^67^. The algorithm searches for *trans* target genes is based on mRNA sequence complementary and RNA duplex energy prediction, assessing the binding of lncRNA and mRNA molecules. The target sequences and complementary energy between lncRNAs and targets were analyzed by the BLAST algorithm and RNAplex (-e -60), respectively^68^.

All candidate lncRNAs were used to predict miRNA target mimic sites using psRoboot with default parameters^69^. The miRNA target mimicry from *B*. *rapa* lncRNAs were predicted according to the rules in previous study^33^. Putative target genes of miRNAs were predicted *in silico* using psRNATarget^70^.

### Co-expression network construction

A weighted gene co-expression network of differentially expressed mRNAs was constructed using the WGCNA package in R^71^. An unsupervised co-expression relationship was built based on the adjacency matrix which represents the network connection strength between gene pairs. The one-step network construction option with a soft-thresholding power value of 10, min Module Size = 30 and merge Cut Height = 0.25 were used. The other parameters were set to default values. And the *q*-values (false discovery rate, FDR) were calculated to detect the significant (*q*-value < 0.05). Highly similar modules were subsequently identified by clustering and then merged together to new modules on the bias of eigengenes. The correlation of each module was also analyzed and visualized by a heatmap.

To identify key genes related to heat tolerance, we constructed a co-expression network of the interactions between the differentially expressed lncRNAs and mRNAs based on *cis*- or *trans*-regulation. For each pair of lncRNAs and mRNAs, the Pearson correlation coefficient (PCC) of expression patterns were calculated using the expression values^72^. Using the STRING protein-protein interaction database, the mRNA-mRNA interaction relationship was analyzed. And the lncRNAs or mRNAs as the target genes of miRNAs were predicted using psRNAtarget^70^. Then, the co-expression network was visualized by Cytoscape software^73^.

### Quantitative real time RT-PCR

For validation of the lncRNAs and mRNAs in Chinese cabbage, total RNA from each sample was extracted using the RNAiso Reagent Kit (TaKara, China). Single-strand cDNA was synthesized with approximately 2 μg RNA from each sample by using the RevertAid First Strand cDNA Synthesis Kit (Fermentas, USA). Using SYBR Premix Ex Taq Kit (TaKaRa, China), the qRT-PCRs were performed on an ABI StepOne Real-Time PCR System (Applied Biosystems, USA). The melting curve was used to verify only one specific product. Three replicates were performed for each sample. *BrActin* was used as the internal gene for mRNAs and lncRNAs. All the primers used in the study are listed in Table S11. The relative expression levels of mRNAs and lncRNAs were normalized using a ^△△^CT method^74^. Significance of the expression level between the sensitive variety ‘GHA’and tolerant variety ‘XK’ were determined by one-way of variance with *t*-rest (*P* < 0.05 or *P* < 0.01).

### URLs

Brassica Database (BRAD), (http://brassicadb.org/brad/). Cytoscape software, http://www.cytoscape.org/. psRNAtarget, http://plantgrn.noble.org/psRNATarget/. AgriGO, http://systemsbiology.cau.edu.cn/agriGOv2/. STRING database, https://string-db.org/. Tophat 2.0 software, http://ccb.jhu.edu/software/tophat/.

## Supplementary information

Supporting Information

Supplementary Files
