# Supplementary material for: Genome-wide analysis of long non-coding RNAs unveils the regulatory roles in the heat tolerance of Chinese cabbage (Brassica rapa ssp.chinensis)
Source: Sci Rep. 2019 Mar 21;9:5002. doi: 10.1038/s41598-019-41428-2 (PMC6428831; doi:10.1038/s41598-019-41428-2)
Supplement: Supplementary file 1 — Supporting Information [file 41598_2019_41428_MOESM1_ESM.pdf]

Supporting Information

Genome-wide analysis of long non-coding RNAs unveils the regulation roles in heat tolerance of Chinese cabbage (*Brassica rapa* ssp.chinensis)

Aihua Wang[1†], Jihong Hu[2†], Changbin Gao[1], Guanglong Chen[3], Bingcai Wang[1], Chufa Lin[1], Liping Song[1], Yi Ding[3], Guolin Zhou[1]*

1 Wuhan vegetable research institute, Wuhan Academy of Agricultural Science & technology, Wuhan 430345, China

2 Oil Crops Research Institute of the Chinese Academy of Agricultural Sciences, Key Laboratory of Biology and Genetic Improvement of Oil Crops, Ministry of Agriculture, Wuhan 430062, China

3 State Key Laboratory of Hybrid Rice, College of Life Sciences, Wuhan University, 430072, Wuhan, China.

† These authors contributed equally to this work.

Fig. S1 Heatmap of the correlation of the 12 samples of Chinese cabbage.

Fig. S2 Clustering dendrogram of differentially expressed genes (DEGs) using weighted gene co-expression network analysis (WGCNA). Twenty modules were constructed in Chinese cabbage leaves under heat stress.

Table S1 Statistics of the Illumina sequencing data in 12 libraries.

Table S2 Identification and characterization of lncRNAs in Chinese cabbage under heat stress.

Table S3 Comparison of lncRNA identification in this study with previous lncRNAs in three *Brassica* species.

Table S4 The expression level of the differentially expressed lncRNA.

Table S5 LncRNAs target mRNAs by *cis* and *trans* acting in Chinese cabbage.

Table S6 GO enrichment analysis of six modules which are significant related to heat stress.

Table S7 The expression patterns of the differentially expressed genes (DEGs) in Chinese cabbage under heat stress.

Table S8 LncRNAs were predicted as targets of miRNAs in Chinese cabbage.

Table S9 LncRNAs were predicted as endogenous target mimics (eTMs) of miRNAs in Chinese cabbage.

Table S10 LncRNAs as potential miRNA precursors in Chinese cabbage.

Table S11 Primers used for quantitative RT-PCR in this study.

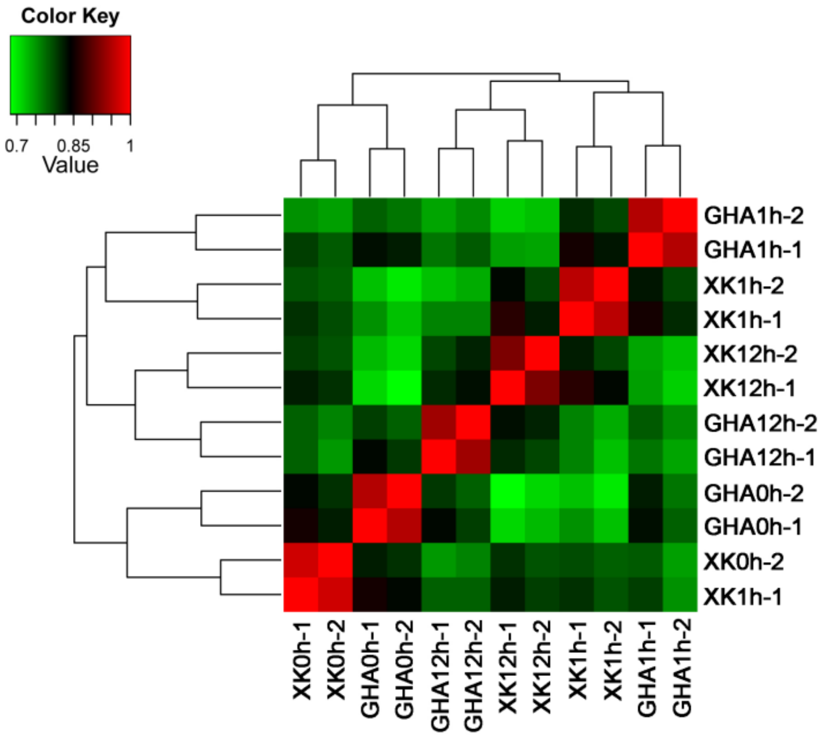

Fig. S1 Heatmap of the correlation of the 12 samples of Chinese cabbage.

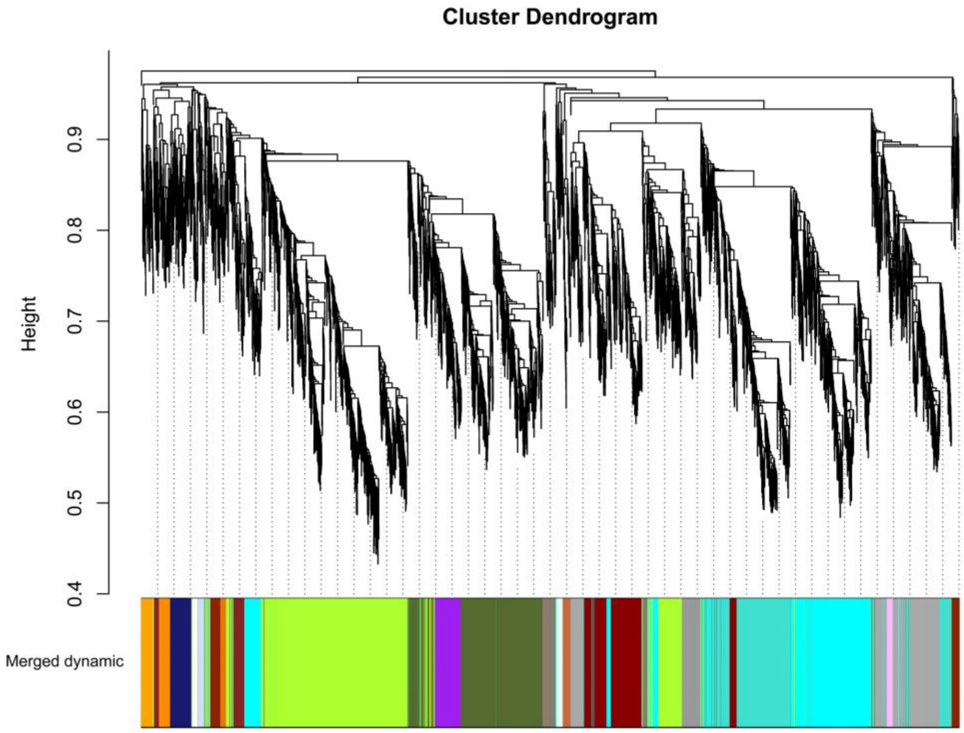

Fig. S2 Clustering dendrogram of differentially expressed genes (DEGs) using weighted gene co-expression network analysis (WGCNA). Twenty modules were constructed in Chinese cabbage leaves under heat stress.
